# Deep mucosal healing in ulcerative colitis: how deep is better?

**DOI:** 10.3389/fmed.2024.1429427

**Published:** 2024-08-02

**Authors:** Xin Jin, Yan You, Gechong Ruan, Weixun Zhou, Ji Li, Jingnan Li

**Affiliations:** ^1^Department of Gastroenterology, Peking Union Medical College Hospital, Chinese Academy of Medical Sciences, Beijing, China; ^2^Department of Pathology, Peking Union Medical College Hospital, Chinese Academy of Medical Sciences, Beijing, China

**Keywords:** ulcerative colitis, deep mucosal healing, endoscopic remission, histological remission, clinical practice

## Abstract

Ulcerative colitis (UC), characterized by its recurrent nature, imposes a significant disease burden and compromises the quality of life. Emerging evidence suggests that achieving clinical remission is not sufficient for long-term remission. In pursuit of a favorable prognosis, mucosal healing (MH) has been defined as the target of therapies in UC. This paradigm shift has given rise to the formulation of diverse endoscopic and histological scoring systems, providing distinct definitions for MH. Endoscopic remission (ER) has been widely employed in clinical practice, but it is susceptible to subjective factors related to endoscopists. And there’s growing evidence that histological remission (HR) might be associated with a lower risk of disease flares, but the incorporation of HR as a routine therapeutic endpoint remains a debate. The integration of advanced technology has further enriched the definition of deep MH. Up to now, a universal standardized definition for deep MH in clinical practice is currently lacking. This review will focus on the definition of deep MH, from different dimensions, and analyze strengths and limitations, respectively. Subsequent multiple large-scale trials are needed to validate the concept of deep MH, offering valuable insights into potential benefits for UC patients.

## 1 Introduction

**Figure 1:**
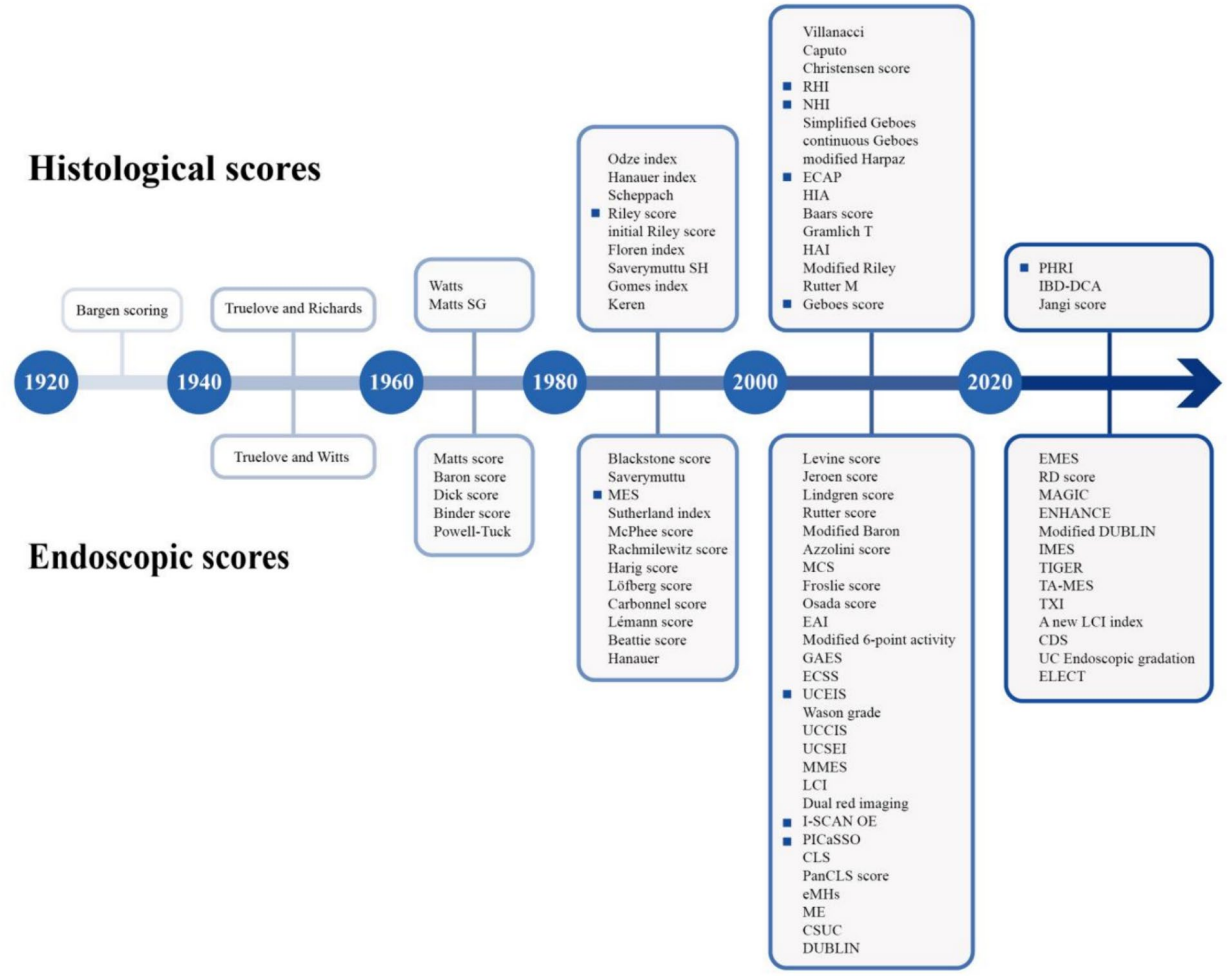
Timeline of endoscopic scores and histological scores (highlighted as the crucial scores discussed in the review).

Ulcerative colitis (UC) is a chronic inflammatory disorder characterized by persistent mucosal inflammation of the colon and rectum. In 2023, the global prevalence of UC is estimated to be 5 million cases, with a continuing upward trend in the incidence (1). The clinical course is hallmarked by recurrent exacerbations and remissions, which can occur spontaneously or prove refractory to therapeutic interventions (2). Although overall mortality in UC patients may not significantly differ from that of the general population (3), chronic inflammation can confer an elevated risk of hospitalizations, surgeries, colorectal cancer, and compromised quality of life. Thus, achievement of remission represents a dual short-term and long-term target. The exploration of remission has expanded along two dimensions, generally, the extent and depth. For optimal outcomes, the treatment strategy in UC has evolved into a “treat to target” paradigm, with the ultimate goal of achieving mucosal normalization. This paradigm has been translated into the concept of “mucosal healing (MH),” which was initially defined by endoscopic remission (ER) in clinical trials and routine clinical practice. However, mounting evidence showed the persistence of histological inflammation in some patients who have achieved ER. There have been suggestions to incorporate histological remission (HR) as a component of the MH criteria. As shown in Figure 1, various endoscopic and histological scores have emerged, yet no universally accepted definition of ER or HR has been established. Recently, technological advancements have introduced instruments capable of providing more precise and real-time assessments, which force reevaluating and refining the definition of MH.

The optimal definition of MH has been the subject of debate for more than six decades, and a universally validated definition continues to be elusive. In addition, the concept of deep MH has recently been proposed to indicate more reliable remission, as a new treatment target. In this review, we aimed to provide an extensive overview of MH in UC and to critically evaluate the strengths and limitations of various criteria employed in clinical practice.

## 2 Endoscopic remission

The foundation for selecting therapeutic targets relies on the assessment of underlying inflammation visible during endoscopy. Various endoscopic scores and indices have been proposed for this purpose (refer to Supplementary Table S1). Key evaluation criteria encompass reproducibility, responsiveness, or sensitivity to change. The quest for the optimal cutoff for MH has been extensively debated.

### 2.1 White light endoscopy based

#### 2.1.1 Mayo endoscopic subscore (MES)

In 1937, Bargen JA described five stages of sigmoidoscopic observation of “chronic ulcerative colitis,” characterized by periods of exacerbation and remission, while they defined it as an infectious disease (4). So it is generally accepted Truelove SC was the first to establish an association between initial endoscopic findings and the clinical status of UC patients, demonstrating that well-treated patients consistently exhibited improvements in endoscopic assessment (5). To quantify mucosal lesions, MES was proposed in 1987, as a prevalent tool in clinical practice over an extended period. Initially, there was a consensus regarding the definition of MH as MES ≦1, characterized by normal mucosa or mucosal erythema, reduced vascular pattern, and mild friability. Subsequently, disputes emerged, as clinical relapses remained not infrequent, even in cases categorized as MES 0. And there is an increasing interest in achieving the more rigorous goals of ER (MES 0, indicative of normal mucosa). Multiple studies have compared the prognosis of MES 0 and MES 1 (partially shown in Table 1). A meta-analysis indicated that patients with MES 0 had a lower risk of clinical relapse (29). Research has suggested MES 0 patients may have reduced risks of requiring escalated therapy, colectomy, and hospitalization (30). In 2021, the updated STRIDE II guidelines recommended that endoscopic healing should be indicated by MES 0 (31).

**Table 1:** Mayo endoscopic score and clinical relapse.

Year	Research	Type	Patients	Intervene	MES 0 vs. MES 1	Follow-up	Which is better
2011	López (6)	Prospective	20	Monotherapy with AZA or MP	MES 0 = 10 (50%); MES 1 = 3 (15%)	27.1 months, range (5–59)	MES 0
2013	Yokoyama (7)	Retrospective	24	——	MES 0 = 9 (37.5%); MES 1 = 15 (62.5%)	60 months	MES 0
2015	Ikeya (8)	Retrospective	29	Tacrolimus	Unrevealed	57.6 weeks	No significant difference.
2016	Barreiro-de Acosta (9)	Prospective	187	Maintenance treatment	MES 0 = 126 (67.3%); ES 1 = 61 (32.7%)	12 months	MES 0
2016	Boal Carvalho (10)	Retrospective	138	——	MES 0 = 61 (44.2%); MES 1 = 77 (55.8%)	12 months	MES 0
2016	Jae Hyun Kim (11)	Retrospective	215	——	MES 0 = 113 (52.6%); MES 1 = 87 (40.4%)	80 (12–118) months	MES 0
2016	Takuya Yoshino (12)	Retrospective	88	Maintenance treatment	MES 0 = 43 (48.9%); MES 1 = 45 (51.1%)	1.2 years (range, 0.02–5.20)	No significant difference.
2016	Asuka Nakarai (13)	Retrospective	194	——	MES 0 = 94 (49%); MES 1 = 57 (29%)	unrevealed	MES 0
2017	Ponte (14)	Retrospective	60	——	MES 0 = 32 (53.3%); MES 1 = 28 (46.7%)	0–72 months	MES 0
2017	Giuseppe Frieri (15)	Prospective	52	The high-dose mesalazine regimen consisted of ⩾ 3.6 g/day orally plus 1–4 g/day topically for the first 6 months after steroid withdrawal, and subsequent reduction of the frequency of administration of the topical therapy to 3 times/week.	MES 0 = 29 (55.77%); MES 1 = 17 (32.7%)	3 years	MES 0
2018	Narang (16)	Prospective	46	A stable dose of mesalamine and azathioprine/6-mercaptoprine	MES 0 = 36 (78.3%); MES 1 = 10 (21.7%)	18 months	No significant difference.
2018	Takayuki Yamamoto (17)	Prospective	164	Induction therapy and remission maintenance	MES 0 = 84 (51%); MES 1 = 80 (49%)	12 months	No significant difference.
2018	Triana Lobatón (18)	prospective	96	Maintenance treatment	MES 0 = 63 (66%); MES 1 = 33 (34%)	12 months	No significant difference.
2019	Mimari Kanazawa (19)	prospective	166	Maintenance treatment	MES 0 = 91 (54.8%); MES 1 = 75 (45.2%)	unrevealed	MES 0
2020	Marietta Iacucci (20)	prospective	307	Maintenance treatment	MES 0 = 168 (54.7%); MES 1 = 47 (15.3%)	12 months	MES 0
2021	Taku Kobayashi (21)	Prospective	56	Discontinued infliximab	MES 0 = 32 (69.6%); MES 1 = 14 (30.4%)	48 weeks	MES 0
2021	Mark T Osterman (22)	Prospective	100	Maintenance treatment	MES 0 = 5 (5%); MES 1 = 56 (56%)	12 months	MES 0
2022	Ge Chong Ruan (23)	Prospective	63	Maintenance treatment	MES 0 = 13 (20.6%); MES 1 = 13 (20.6%)	23.5 (16.25–27.75) months	MES 0
2022	Yosuke Shimodaira (24)	Retrospective	102	——	MES 0 = 41 (40.2); MES 1 = 26 (25.5)	median 103.9 weeks	MES 0
2022	Cristian Hernández-Rocha (25)	Retrospective	113	——	MES 0 = 46 (40.7%); MES 1 = 43 (38.1%)	unrevealed	No significant difference.
2023	Gyeol Seong (26)	Retrospective	492	——	MES 0 = 253 (51.4%); MES 1 = 239 (48.6%)	549 days	MES 0
2023	Yukie Hayashi (27)	Prospective	146	Maintenance treatment	MES 0 = 44 (30.1%); MES 1 = 102 (69.9%)	232.0 ± 91.9 days	MES 0
2023	Natsuki Ishida (28)	Retrospective	75	Maintenance treatment	MES 0 = 43 (57.3%); MES 1 = 32 (42.7%)	unrevealed	MES 0

However, when the adopting MES in clinical practice, inflammatory bowel disease (IBD) physicians should take into account disease extent. Boal et al. reported that in the subgroup of patients with left-sided or extensive colitis, MES 1 had a higher risk of relapse (29.7% vs. 11.1%, *p* = 0.049), whereas this distinction was not observed in those with proctitis (25.0% vs. 12.0%, *p* = 0.202). These findings suggested achieving deep MH may hold greater importance for patients with extensive lesions (10). In contrast to the final result equal to the maximum MES, considering the disease extent, the modified Mayo endoscopic score (MS) aggregates MESs of 5 colonic segments to form a 15-point scale (32). And on the basis of this, Lobaton T et al. multiplied MS by the maximal extent of inflammation to calculate the extended modified score (EMS), and proposed the modified Mayo endoscopic score (MMES), obtained by dividing EMS by the number of active segments. They demonstrated that MMES >0.8 could accurately predict active histological activity (33). This approach provides guidance for a more accurate method of recording disease activity without creating a new scoring system. And conducting long-term trials is imperative to thoroughly evaluate its predictive value.

#### 2.1.2 Ulcerative colitis endoscopic index of severity (UCEIS)

The UCEIS is another well-validated index employed in different studies with satisfactory interobserver reliability and reproducibility (34). It comprises three descriptors—vascular pattern, bleeding, erosions and ulcers, yielding a total score ranging from 0 to 8. Due to elaborate standards, UCEIS has the capability of recording changes in mucosal status throughout the disease course. A study has demonstrated that clinical information has minimal effect on assessment outcomes, implying that UCEIS is valuable for predicting outcomes of patients with clinical remission in clinical routine practice (35, 36). Travis et al. first stringently defined UCEIS-ER as a score of 0 for all three descriptors, allowing blurring or loss of capillary margins with a recognizable vascular pattern, no visible bleeding, and no erosions or ulceration (37). In subsequent studies, UCEIS ≤1 was regarded as an indicator of MH. Patients with UCEIS ≤1 exhibited an overall event-free survival rate of 93.3% at 6 months and 1 year, 81.6% at 2 years, and 65.3% at 3 years (38). An international organization for the study of inflammatory bowel disease (IOIBD) once voted for ER, with UCEIS 0 and UCEIS ≤1 ranking the first and the third criteria (39). In 2021, the updated STRIDE II guidelines also recommended that ER should be indicated by an UCEIS ≤1. Further studies are required to establish thresholds.

#### 2.1.3 Others

Baron Score, a utilized score, was initially developed for sigmoidoscopy assessment. It mainly described vascular pattern and bleeding. A series of derivative scores were generated. Feagan refined Baron Score into a detailed classification, and defined ER as modified Baron score ≤ 1 (40, 41). As for Rachmilewitz endoscopic subscores, an inactive state was defined as 0–3 (42). To reveal the inflammation of the entire colon, Samuel et al. established ulcerative colitis colonoscopic index of severity (UCCIS) based on a full colonoscopy (43). Compared with UCEIS, UCCIS incorporates granularity into grading items. It was reported that the optimal cut-off values of UCCIS for patients who experienced relapse within 2 years and 5 years were 9.8 and 10.2, respectively (44). And it holds potential as a useful tool to predict mid-to long-term clinical relapse. However, these scoring systems are still needed to be validated externally and evaluated in real-world studies.

### 2.2 Virtual chromoendoscopy (VCE) based

With the rapid development of cutting-edge technology, image-enhanced endoscopic techniques have gained widespread utilization. These can be categorized into two primary types, dye chromoendoscopy (DCE) and VCE, which are specialized in enhancing the visualization of mucosal surface, blood vessels, and color tones. Compared to DCE, VCE has distinct advantages in terms of cost-and time-efficiency, and meanwhile VCE based ER could more accurately predict the HR compared to white-light endoscopy (WLE) (45). The new generation of VCE modalities mainly encompasses: narrow band imaging (NBI, Olympus), the I-SCAN or OE (Pentax), blue laser image (BLI, Fujifilm), and linked color imaging technology (LCI, Fujifilm).

#### 2.2.1 Paddington international virtual chromoendoscopy score (PICaSSO)

In pursuit of a more precise definition of MH, Iacucci et al. employed VCE to develop the PICaSSO scoring system with I-SCAN. This system assessed subtle mucosal and vascular features, beyond absence of inflammatory lesions and ulcers. It has demonstrated a strong correlation with histological scores, superior to MES and UCEIS (*p* < 0.01). PICaSSO-ER was defined as PICaSSO ≤3. It showed that PICaSSO-ER could predict better outcomes at both 6-month and 12-month follow-up (HR 0.19 (0.11–0.33) and 0.22 (0.13–0.34), respectively) (20, 46). Remarkably, they proved the difference in clinical outcomes between MES 1 over MES 0 was greater than PICaSSO 4–8 over PICaSSO ≤3. And it has been externally validated in our previous long-term prospective study (23). A multicenter international study has provided robust evidence that the PICaSSO could be consistently generated with multiple platforms, including NBI and LCI/BLI, with an interclass correlation coefficient (ICC) of 0.825. The accuracy rates for NBI, LCI, and BLI were 0.808, 0.827, and 0.79 (46). However, the PICaSSO is still not well practiced worldwide and the further large-scale and long-term follow-up studies are needed to elucidate its potential in clinical practice.

#### 2.2.2 I-SCAN optical enhancement scoring systems

I-SCAN OE system, integrating digital and optical enhancements, is capable of detecting more intricate mucosal and vascular patterns. Different from conventional UC description as “loss of vascular pattern,” the system identifies features such as spiral isolated, crowded tortuous, and irregular vessels. It is reported that I-SCAN OE was significantly correlated with MES, and approximately 31–41% of MES 0 patients still exhibited abnormal mucosal or vascular patterns detected by I-SCAN OE (47). Recently, the mucosal analysis of inflammatory gravity by i-scan TE-c image (MAGIC) score has been introduced. It further quantified the degree of inflammation by correlating the value with the reference value for each pixel in the hue/saturation/brightness color space. The MAGIC score of the MES 1 group was significantly higher than that of the MES 0 group (*p* = 0.0034), indicating its potential value for evaluating mucosal inflammation in clinically quiescent patients. It is going to be a competitive tool for clinical practice, as it offers more comprehensive assessment throughout colonic mucosa, unlike biopsy which only represents a part of colon (48). However, there remains some uncertainty regarding whether I-SCAN scores can be classified as endoscopic scores, because I-SCAN scores and WLE endoscopic scores do not measure exactly identical parameters.

### 2.3 Other novel endoscopic technology

The challenges of distinguishing mild from quiescent disease, coupled with the gap between ER and HR, have impeded the widespread clinical application of MH. Advancements in equipment, rendering enhanced capabilities for detecting subtle inflammation, have the potential to address these issues. Surpassing the standard-definition WLE, which often causes underestimation of mucosal lesions, high-definition WLE and magnified endoscopy provide image signals with higher pixel density (49). Red dichromatic imaging (RDI) enables the identification of blood vessels within the deeper layers of mucosa and submucosa. It has shown a closer correlation with histology than WLE scoring systems (50). Confocal laser endomicroscopy (CLE) is considered as having the highest resolution. Gheorghe et al. established and validated endomicroscopic mucosal healing score (eMHs), defining eMHs <1 as complete MH, with a sensitivity of 100% (95% CI 15.81–100%), a specificity of 93.75% (95% CI 69.77–99.84%), and an accuracy of 94.44% (51). CLE can also assess the integrity of the intestinal barrier, with high accuracy in predicting the disease’s future course, superior to MES 0 (52). Assessed by endocytoscopy, the ErLangen endocytoscopy in colitis score (ELECT), predicted HR with an accuracy of 91.3%, along with a sensitivity and specificity of 88% and 95.2% (53).

Even with experienced endoscopists and advanced endoscopy, interobserver variability, and subjectivity cannot be ignored. To enhance accuracy and minimize the bias, phase 3 trials favor a “2 + 1” central reads approach. If two central readers reach a consensus, the score is considered as final; otherwise, a third blinded central reader is engaged, often through voting or averaging (54). However, this approach comes at the cost of significant operational delays and expenses. To meet real-world efficiency and break the limits of expert requirement for defining MH, several artificial intelligence-assisted endoscopic systems have been developed. Bossuyt P et al. established and validated the first objective operator-independent endoscopic scoring system based on red density (RD). The algorithm provided automated redness assessment by integrating pattern recognition and proved to correlate with other endoscopic scores (*p* < 0.0001) (55). Takenaka et al. constructed a deep neural network for the evaluation of UC (DNUC), which achieved an accuracy ratio of 90.1% (95% CI 89.2–90.9%) for identifying ER and 92.9% (95% CI 92.1–93.7% for HR (56). Huang et al. developed a computer-aided diagnosis system with deep learning and machine learning (DLML-CAD), demonstrating performance comparable to IBD endoscopists and superior to non-IBD and trainee endoscopists. It achieved an accuracy of 94.5% for MES 0–1 and 89.0% for MES 0 (57). Takabayashi K et al. applied a ranking-convolutional neural network to the UC Endoscopic Gradation Scale (UCEGS), and reported that UCEGS accurately represented the assessment of the endoscopic severity by IBD expert endoscopists (58).

### 2.4 Strengths and limitations

According to consensus guidelines, ER is recommended for both clinical practice and clinical trial endpoints in UC beyond clinical remission, owing to its potential to mitigate the risk of recurrence and post-treatment complications. Nonetheless, it is essential to acknowledge the inherent limitations of ER. Firstly, the application of endoscopy is an invasive, costly, and time-consuming procedure, so that compliance is still a challenge. Secondly, there remains a gap between achieving ER and the attainment of complete remission. A systematic review has summarized the rates of clinical relapse for MES 1 patients ranged from 8% to 66.7%, and for MES 0 patients from 0 to 33.3% (29). Thirdly, the scoring of endoscopic findings is currently influenced by the expertise and subjective judgment of endoscopists. It was reported that interobserver agreement concerning endoscopic scores was moderate, despite the potential for improvement through specialized training, resulting in an increase from κ 0.51 (95% CI, 0.48–0.55) to 0.76 (95% CI, 0.72–0.79) (59). Fourthly, many newly developed endoscopic scores still need more external validation and long-term follow-ups. Consequently, the definition of deep MH based on the endoscopic scoring index is always a dynamic and developing concept majorly depending on evidence from the future valuable studies.

## 3 Histological remission

Multiple histological scoring indices, similar to endoscopic scores, have been developed to assess disease status, with a validated close correlation between histological scores and clinical scores. HR has garnered significant attention for its potential to detect deep MH, especially considering the presence of active histologic findings in patients with ER.

### 3.1 Geboes index (GS)

GS represents a widely utilized histological grading system. It encompasses six primary grades: Grade 0, structural change only; Grade 1, chronic inflammation; Grade 2, lamina propria neutrophils; Grade 3, neutrophils in epithelium; Grade 4, crypt destruction; and Grade 5, erosions or ulcers, with the capacity for further refinement within each grade (60). The definition of GS-HR has not achieved universal validation yet. Several studies have proposed HR to be defined by GS <3.1, with the absence of neutrophils in epithelium (61, 62). Other studies defined HR as GS ≤2.0, signifying no increase in neutrophils or eosinophils in lamina propria (63). Cushing’s study focused on ER patients, and observed that complete histological normalization, defined as GS = 0, significantly reduced the likelihood of relapse, while resolution of active inflammatory infiltrate (GS ≥3.1, GS ≥2.1a, and GS ≥2.1b) were not associated with risk of relapse. It suggested that histological activity plays a pivotal role as a prognostic determinant in the cohort of patients with ER (64). More recently, the GS has been converted into a continuous scale for convenience, calculated by summing the numerical values of the various sub-scores, yielding between 0 and 22. But this has seldom been used and validated in clinical practice (65).

### 3.2 Riley index

The Riley index primarily focuses on the evaluation of the density and distribution of neutrophils, as well as the assessment of mucosal defects. It consists of acute inflammatory cell infiltrate, mucin depletion, chronic inflammatory cell infiltrate, surface epithelial integrity, crypt architectural irregularities and crypt abscess (66). It was reported that the Riley subcomponent, architectural irregularity ranked as the most predictive factor for clinical relapse (22). Riley-HR criteria remains ongoing investigation. Riley 0–1 and Riley 0 as HR were once adopted in different studies (67, 68).

### 3.3 Robarts histopathology index (RHI)

RHI stands as a validated tool for assessing histopathological activity assessment. RHI ranges from 0 to 33, and is derived from the evaluation of four parameters: chronic inflammatory infiltrate, lamina propria neutrophils, neutrophils in the epithelium, erosion, and ulceration. Initially proposed by Mosli et al., RHI-HR was defined as RHI ≤6 (69). Up till now, HR is commonly defined as RHI ≤3, with sub-scores of 0 for epithelium and lamina propria neutrophils. Fernando’s investigation demonstrated RHI ≤3 had a high positive predictive value (PPV = 95%) for HR (according to GS) (65). For patients with RHI ≤3, the rates of clinical relapse or therapeutic escalation at 6 and 12 months were 11.7% and 15.9%. More strictly, some studies defined HR as RHI ≤1, indicating the complete absence of mucosal neutrophils and basal plasmacytosis. This rigorous definition is infrequently employed, due to its challenging attainment in clinical practice (70).

### 3.4 Nancy histology index (NHI)

NHI is another recently developed and validated score that has gained recognition for its simplicity. NHI is based on three key items: ulceration, acute inflammatory cells infiltrate and chronic inflammatory cells infiltrate, which account for most of the disease activity. It has demonstrated good intrareader reliability and interobserver reliability (ICC = 0.880 and 0.865). For HR, NHI <2 was voted as an appropriate threshold (71), refering to the absence of neutrophils, and allowing for chronic inflammatory infiltrate, including lymphocytes and/or plasmocytes and/or eosinophils in lamina propria (72). In a prospective observational multicenter study, it has been corroborated that NHI <2 predicted a reduced likelihood of steroids use (OR 0.26, 95% CI 0.11–0.56, *p* = 0.002) and hospitalization (OR 0.30, 95% CI 0.12–0.76, *p* = 0.01) (73). In D′Amico’s study, HR was rigorously defined as NHI =0. Patients with histological disease activity experienced a notably higher rate of colorectal surgery (14% vs. 0%, *p* = 0.01) and hospitalization (36% vs. 7.1%, *p* = 0.001) (74). Given high applicability and consistency with other histological indices, NHI has been widely accepted (75).

### 3.5 PICaSSO histologic remission index (PHRI)

Gui et al. proposed PHRI, offering a simplified dichotomous approach, which exclusively focuses on the presence of neutrophils. They defined HR as PHRI = 0, with fewer negative outcomes (48.65% vs. 13.91%, *p* < 0.00001), and identified a cut-off value of 1 as the best predictor of relapse at 12 months (76). Externally validated by a study enrolling 192 UC patients, PHRI demonstrated risk stratification for relapse that was comparable to established indices, RHI and NHI (*p* > 0.05). Due to its simplicity, PHRI, neutrophil-only assessment, is likely to be an optimal alternative to existing histological scores (77, 78).

### 3.6 Other histological scoring systems

In order to capture subtle histological abnormalities, a structured ECAP (Extent, Chronicity, Activity, Plus additional findings) system was established for MES 0 patients with abnormalities visible on i-Scan imaging. And ECAP-HR was defined as ECAP ≤4 (79). Harpaz histological scoring system (HSS) is one of the easiest scores to use. It employs a validated four-point scale to grade cryptitis, ulceration and erosion (80). Gramlich index is based on infiltrate of neutrophils into the crypt epithelium, and rare neutrophils infiltrating crypt epithelial cell was categorized as mild activity (81). Obviously, it is inevitable that arbitrary visual estimate of percentage value is somewhat subjective. Similar to the development of ER, technology-assisted data input emerged. Gottlieb et al. designed and validated computer-aided diagnosis (CAD) systems for evaluation of UC biopsies, distinguishing HR with a sensitivity and specificity of 89% and 85% (PHRI), 94% and 76% (RHI), and 89% and 79% (NHI) (82).

### 3.7 Strengths and limitations

Achieving HR not only correlates with a diminished risk of disease flares, but holds promise for reducing medication requirements, implying that HR might serve as a valuable indicator for relapse-free outcomes (83). A meta-analysis demonstrated that HR was associated with 20% reduction in relative risk of clinical relapse or disease exacerbation, compared with clinical remission and ER (84). However, there remains debates surrounding the question whether histological healing should be incorporated as a routine therapeutic endpoint. Firstly, in comparison to ER, acquisition of tissue biopsies exerts additional procedural burden, especially for patients already achieving ER, which makes it risky to repetitively conduct such examinations at short intervals. Secondly, there is a lack of standardization in biopsy collection protocols and histopathology description, which presents challenges in interpreting HR results. Some prefer to take distal biopsies, while others opt for sampling from the most inflamed segment observed. Furthermore, there is a heterogeneous distribution of residual inflammation, especially in treated UC, which might lead to an underestimation of disease activity. It also remains uncertain whether biopsies from local sites could reflect the activity of the entire intestinal system. A study examined that segmental normalization did not signal improved clinical outcomes, unlike complete histological normalization (defined as normal mucosa by biopsy in all bowel segments, *p* = 0.008) (85). Thirdly, the scarcity of large-scale research address the long-term risks and benefits of achieving HR concerning the extension of the treatment or more intensive therapy. It is principally because of the absence of medications with proven efficacy in inducing HR (86). Fourthly, the inter-observer variance of HR, as well as ER is unavoidable on account of subjectivity. These complex considerations underscore the need for ongoing research and consensus-building to refine the role of HR as a therapeutic endpoint in clinical practice.

## 4 Combined ER and HR

Taking into account the benefits in both endoscopic and histological scoring systems, there are opinions suggesting that histological assessment of the colonic mucosa based on endoscopic evaluation may provide additional insights into relapse-free survival, as endoscopy and histology serve as complementary tools. A meta-analysis involving 1,360 patients revealed that nearly 30% of patients with endoscopic and clinical remission still exhibited histological activity, and the addition of HR provided enhanced prognostic utility (84). Studies have discovered that histo-endoscopic remission is associated with a mucosal transcriptional profile resembling that of healthy mucosa. Notably, genes and pathways related to UC pathogenesis and prognosis remains activated in patients who only achieve ER (25). However, there is no widely accepted standard definition of combined ER and HR. Hernández et al. founded patients achieving histo-endoscopic remission (GS ≤3.1 and MES ≤1) had a significantly lower risk of relapse (25). And based on UNIFI phase 3 UC clinical studies of ustekinumab, Li K et al. found the achievement of histo-endoscopic MH after induction therapy confered lower disease activity at the end of maintenance therapy than endoscopy and histology alone (87). Carlsen defined deep remission based on MES 0 and GS ≤1 (88). Nardone et al. reported survival advantages for UCEIS ≤1 combined with NHI ≤1 compared to UCEIS ≤1 alone (HR 0.30, 95%CI 0.12–0.75, *p* = 0.02) at the 12-month mark (89). More stringently. Verstockt B et al. established histo-endoscopic mucosal remission (HEMR), combining MES 0, UCEIS 0, and NHI 0, and patients with HEMR was associated with reduced IBD disability (*p* < 0.001) (90). Recently, there has been a proposal of a concept known as “disease clearance” for UC, defined as concurrent achievement of clinical, endoscopic, and histological remission (31). Some researchers also suggested that physical functioning, mental health, and work activity should be included (91, 92). A multi-center retrospective cohort study demonstrated that UC patients with early disease clearance had a reduced risk of hospitalization and surgery (log-rank *p* < 0.0001 and *p* < 0.001) (93). IOIBD has achieved a consensus on defining disease clearance as composition of partial May a score 0, MES 0, and NHI 0. Nevertheless, further prospective trials are acquired and it may evolve in the future (94).

Conversely, there were no differences in relapse-free survival between PICaSSO-ER combined with HR and PICaSSO-ER alone (PICaSSO ≤3 + RHI ≤3 vs. PICaSSO ≤3, *p* = 0.1) (89). In Parigi’s study, they found the stratification of prognostic value by combining ER and HR did not improve outcomes significantly, compared with assessment individually (77). A *post hoc* analysis also demonstrated histo-endoscopic improvement (MES ≤1 and GS <3.2) did not provide additional prognostic value at 1-year follow-up on endoscopic improvement over ER alone (95). The cost-effectiveness and feasibility of targeting disease clearance in the long term within clinical practice remains unknown. Many experts reached a consensus that MH defined as endoscopic improvement and histologic remission should be used as a secondary endpoint (96). Furthermore, there was a significant disparity between estimates and real-world data, which can not be ignored. A multi-center retrospective study showed most of UC patients even did not achieve composite clinical remission and ER in clinical practice (*p* < 0.001) (97). It might be of the greatest resistance on the road to achieve universal both ER and HR.

## 5 Prospective and summary

Beyond ER and HR, many other markers for MH have been explored, including the well established fecal calprotectin (98), and other potential biomarkers (99–106). However, no noninvasive markers could really replace endoscopic and histologic evaluation for the definition of MH.

The attainment of deep MH in UC patients represents the endpoint of “treat to target” approach, with the expectation of extending periods of remission, reducing the necessity for extensive medical interventions, and optimizing therapeutic outcomes. It is imperative to reach a consensus about how to conceptualize “deep remission.” Existing definitions often comprise clinical remission, ER, HR individually, or various combinations thereof. They have different strengths and limitations, and new technology aids in bridging the gap to deep MH. It is essential to recognize that we cannot transfer definitions adopted in clinical trials to daily clinical practice seamlessly, which might be more exploratory. Further large-scale trials are needed to validate the concept of deep MH, assessing the effectiveness and reproducibility, to offer valuable insights into real-world applications and potential benefits for UC patients.

## Author contributions

XJ: Conceptualization, Writing – original draft. YY: Conceptualization, Writing – original draft. GR: Writing – review & editing. WZ: Writing – review & editing. JiL: Funding acquisition, Supervision, Validation, Writing – review & editing. JinL: Writing – review & editing.

## Glossary

UC: Ulcerative colitis
MH: Mucosal healing
ER: Endoscopic remission
HR: Histological remission
WLE: White light endoscopy
MES: Mayo endoscopic subscore
MMES: modified Mayo endoscopic score
UCEIS: Ulcerative colitis endoscopic index of severity
UCCIS: Ulcerative colitis colonoscopic index of severity
VCE: Virtual chromoendoscopy
DCE: Dye chromoendoscopy
OE: Optical enhancement
CLE: Confocal laser endomicroscopy
CI: Confidence interval
GS: Geboes index
RHI: Robarts histopathology index
NHI: Nancy histology index
OR: Odds ratios
PHRI: PICaSSO histologic remission index
ECAP: Extent, Chronicity, Activity, Plus additional findings
