# Supplementary material for: Deep mucosal healing in ulcerative colitis: how deep is better?
Source: Front Med (Lausanne). 2024 Aug 2;11:1429427. doi: 10.3389/fmed.2024.1429427 (PMC11327023; doi:10.3389/fmed.2024.1429427)
Supplement: Supplementary file 1 [file Table_1.DOCX]

**Supplementary material**

**Supplementary Table 1** Endoscopic scores of ulcerative colitis

| Year | Scores | Range | ER |
| --- | --- | --- | --- |
| 1937 | Bargen Scoring[1] | 0-4 | 0 |
| 1955 | Truelove and Witts Endoscopy Index[2] | 1-3 | 1 |
| 1961 | Matts score[3] | 1–4 | <2 |
| 1964 | Baron score[4] | 0-3 | ≤ 1 |
| 1964 | Dick score[5] | 0-4 | 0 |
| 1970 | Binder score[6] | 1-4 | undefined |
| 1978 | Powell-Tuck Index[7] | 0-2 | 0 |
| 1984 | Blackstone score[8] | 1-8 | 1-2 |
| 1986 | Saverymuttu score[9] | 0-12 | undefined |
| 1987 | Mayo endoscopic subscore[10] | 0-3 | 0, 0-1 |
| 1987 | Sutherland Index[11] | 0-3 | ≤ 1 |
| 1987 | McPhee score[12] | 0-3 | 0 |
| 1989 | Rachmilewitz Endoscopic score[13] | 0–12 | 0-4 |
| 1989 | Harig score[14] | 0-10 | 0 |
| 1994 | Löfberg Score[15] | 0-3 | 0 |
| 1994 | Carbonnel score[16] | severe、moderate | undefined |
| 1995 | Lémann score[17] | 0-3 | 0 |
| 1996 | Beattie score[18] | 0-3 | 0 |
| 1998 | Investigators Global Evaluation/Sigmo idoscopic Inflammation Grade Score[19] | 0-3 | 0 |
| 2002 | Levine score[20] | normal、mild、moderate、severe | normal or mild |
| 2002 | Jeroen Score[21] | 0-12 | 0-3 |
| 2002 | Lindgren score[22] | 0-3 | 0-1 |
| 2003 | Rutter score[23] | 0-4 | undefined |
| 2005 | Modified Baron Score[24] | 0–4 | ≤1 |
| 2005 | Azzolini Score[25] | 0-30 | undefined |
| 2006 | ,Magnifying colonoscopy (MCS) grades[26] | 1-4 | undefined |
| 2007 | Froslie score[27] | 0-2 | 0-1 |
| 2008 | Osada score[28] | 0-21 | 0 |
| 2010 | Endoscopic activity index (EAI)[29] | 0-16 | undefined |
| 2010 | Modified 6-point Activity Index[30] | 1-6 | 1 |
| 2011 | Global assessment of endoscopic severity (GAES)[31] | 0-3 | 0 |
| 2011 | Endocytoscopy system score (ECSS)[32] | 0-6 | undefined |
| 2012 | Ulcerative Colitis Endoscopic Index of Severity (UCEIS) [33] | 0–8 | ≤1 |
| 2012 | Wason grade[34] | 1-3 | 1 |
| 2013 | Ulcerative colitis colonoscopic index of severity (UCCIS)[35] | 162 | ≤9.8 |
| 2014 | Ulcerative Colitis Segmental Endoscopy index[36] | 0-50 | undefined |
| 2015 | Modified Mayo Score (MMES)[37] | 0-25 | ≤0.8 |
| 2017 | Linked Colour Imaging index[38] | A-C | A |
| 2017 | Dual red imaging scores[39] | 1-4 | undefined |
| 2017 | I-SCAN OE[40] | 2-8 | ≤3 |
| 2017 | Paddington International Virtual ChromoendoScopy ScOre (PICaSSO)[41] | 0-15 | ≤3 |
| 2017 | Confocal Leak Score (CLS) [42] | 0-100 | undefined |
| 2018 | Pancolonic Modified Mayo Score[43] | 0-45 | 0-5 |
| 2018 | Confocal Laser Endomicroscopy Mucosal Healing Score for UC (eMHs)[44] | 0-4 | < 1 |
| 2018 | Magnifying Endoscopic Stratification[45] | A-D | A |
| 2018 | Capsule Scoring of Ulcerative Colitis (CSUC)[46] | 0-14 | undefined |
| 2019 | Degree of Ulcerative Colitis Burden of Luminal Inflammation (DUBLIN)[47] | 0-9 | undefined |
| 2020 | Extended Mayo Endoscopic Score (EMES)[48] | 0-55 | undefined |
| 2020 | Red density (RD) system[49] | 0-200 | 60 |
| 2020 | Mucosal Analysis of Inflammatory Gravity by i‐scan TE‐c Image (MAGIC)[50] | 0-10000 | undefined |
| 2021 | Confocal laser ENdomicroscopy for histological HeAliNg in ulCErative colitis index (ENHANCE )[51] | 0-5.5 | undefined |
| 2022 | Modified DUBLIN score[52] | 0-24 | undefined |
| 2022 | Improved Mayo Endoscopic Score (IMES)[53] | 0-5 | 0 |
| 2022 | Toronto IBD Global Endoscopic Reporting [TIGER] Score[54] | per segment: 0-560 | per segment: inactive-mild endoscopic activity : 0–4 points |
| 2022 | Time-adjusted average Mayo endoscopic score (TA-MES)[55] | undefined | undefined |
| 2022 | Texture and color enhancement imaging (TXI)[56] | 0-2 | 0-1 |
| 2023 | A new LCI index[57] | undefined | undefined |
| 2023 | Cumulative Disease Score (CDS)[58] | 0-450 | undefined |
| 2023 | UC Endoscopic Gradation Scale[59] | 0-10 | undefined |
| 2023 | ELECT (ErLangen Endocytoscopy in ColiTis) score[60] | 0-6 | ≤2 |

**Supplementary Table 2** Histological scores of ulcerative colitis

| Year | Scores | Range | HR |
| --- | --- | --- | --- |
| 1956 | Truelove and Richards Index[61] | 0-4 | undefined |
| 1961 | Matts SG[62] | 1-5 | 1 |
| 1966 | Watts[63] | 0-3 | 0 |
| 1984 | Keren[64] | —— | Crypt Changes±Chronic Inflammation |
| 1986 | Gomes Index[65] | 0-4 | undefined |
| 1986 | Saverymuttu SH[66] | 0-12 | undefined |
| 1987 | Floren index[67] | 1-5 | 1 |
| 1988 | the initial Riley Score[68] | 0-4 | undefined |
| 1991 | Riley Score[69] | 0-18 | 0 |
| 1992 | Scheppach[70] | 0-3 | undefined |
| 1993 | Hanauer index[71] | 0-3 | Remission, grade 0 or 1 (clinically normal mucosa or inactive UC), with a minimum of a 1-grade decrease from baseline to final visit |
| 1993 | Odze index[72] | —— | absence of all six features of chronic and no acyove disease. |
| 2000 | Geboes score[73] | 0-5 | 0, ≤2, < 3.1 |
| 2004 | Rutter M[74] | 0-4 | undefined |
| 2005 | Modified Riley Score[75] | 0-7 | undefined |
| 2007 | histological activity index (HAI)[76] | 0-3 | 0 |
| 2007 | Gramlich T[77] | mild, moderate, severe activity | undefined |
| 2012 | Baars score[78] | 0-3 | undefined |
| 2013 | the 6-point histologic inflammatory scale (HIA)[79] | 0-5 | 0 |
| 2015 | Extent, Chronicity, Activity, Plus additional findings (ECAP)[80] | 0-26 | ≤4 |
| 2015 | modified Harpaz Index[81] | 0-3 | 0 |
| 2016 | continuous Geboes Score[82] | 0-22 | ≤6 |
| 2017 | Simplified Geboes Score[83] | 0-4 | 2A |
| 2017 | Nancy Index[84] | 0-4 | 0 |
| 2017 | Robarts Histopathology Score (RHI)[85] | 0-33 | ≤3 |
| 2017 | Christensen score[86] | 1-3 | undefined |
| 2017 | Simplified Histological MH Scheme[87] | 0-8 | ≤1 |
| 2017 | Simplified pathological score[88] | 0-8 | ≤1 |
| 2020 | Jangi score[89] | remission, mild, moderate, severe | remission |
| 2021 | Inflammatory Bowel Disease [IBD]—Distribution [D], Chronicity [C], Activity [A] score [IBD-DCA Score][90] | D0-2,C0-2,A0-2 | histological normalisation [D0, C0, A0 |
| 2022 | PICaSSO Histologic Remission Index (PHRI)[91] | 0-4 | 0 |

**References**

1. Bargen JA. (1937). The medical management of chronic ulcerative colitis: (section of surgery: sub-section of proctology). Proc R Soc Med. 30, 351–362.
2. Truelove S.C. Witts L.J. (1955). Cortisone in ulcerative colitis; final report on a therapeutic trial. Br Med J. 2, 1041-1048.
3. Matts SG. (1961). The value of rectal biopsy in the diagnosis of ulcerative colitis. Q J Med. 30, 393-407.
4. Baron J.H., Connell A.M., Lennard-Jones J.E. (1964). Variation between observers in describing mucosal appearances in proctocolitis. Br Med J. 1, 89–92.
5. Dick A.P., Grayson M.J., Carpenter R.G., Petrie A. (1964). Controlled trial of sulphasalazine in the treatment of ulcerative colitis. Gut. 5, 437-442.
6. Binder V. (1970). A comparison between clinical state, macroscopic and microscopic appearances of rectal mucosa, and cytologic picture of mucosal exudate in ulcerative colitis. Scand J Gastroenterol. 5, 627-632.
7. Powell-Tuck J., Bown R.L., Lennard-Jones J.E. (1978). A comparison of oral prednisolone given as single or multiple daily doses for active proctocolitis. Scand J Gastroenterol. 13, 833-837.
8. Blackstone M.O. (1984). Differentiation ulcerative colitis from Crohn’s disease. In: Blackstone MO, editor. Endoscopic interpretation: normal and pathologic appearance of the gastrointestinal tract. New York: Raven;1984. p. 464–96
9. Saverymuttu S.H., Camilleri M., Rees H., Lavender J.P., Hodgson H.J., Chadwick V.S. (1986). Indium 111-granulocyte scanning in the assessment of disease extent and disease activity in inflammatory bowel disease. A comparison with colonoscopy, histology, and fecal indium 111-granulocyte excretion. Gastroenterology. 90, 1121-1128.
10. Schroeder K.W., Tremaine W.J., Ilstrup D.M. (1987). Coated oral 5-aminosalicylic acid therapy for mildly to moderately active ulcerative colitis. A randomized study. N Engl J Med. 317, 1625-1629.
11. Sutherland L.R., Martin F. (1987). 5-Aminosalicylic acid enemas in treatment of distal ulcerative colitis and proctitis in Canada. Gastroenterology. 32, 1894–1898.
12. McPhee M.S., Swan J.T., Biddle W.L., Greenberger N.J. (1987). Proctocolitis unresponsive to conventional therapy. Response to 5-aminosalicylic acid enemas. Dig Dis Sci. 32, 76S-81S.
13. Rachmilewitz D. (1989). Coated mesalazine (5-aminosalicylic acid) versus sulphasalazine in the treatment of active ulcerative colitis: a randomised trial. BMJ. 298, 82–86
14. Harig J.M., Soergel K.H., Komorowski R.A., Wood C.M. (1989). Treatment of diversion colitis with short-chain-fatty acid irrigation. N Engl J Med. 320, 23-28.
15. Hanauer S, Schwartz J, Robinson M, Roufail W, Arora S, Cello J, et al. (1993). Mesalamine capsules for treatment of active ulcerative colitis: results of a controlled trial. Am J Gastroenterol. 88, 1188–1197.
16. Löfberg R, Ostergaard T.O., Langholz E, Schiöler R, Danielsson A, Suhr O, et al. (1994). Budesonide versus prednisolone retention enemas in active distal ulcerative colitis. Aliment Pharmacol Ther. 8, 623-629.
17. Carbonnel F, Lavergne A, Lémann M, Bitoun A, Valleur P, Hautefeuille P, et al. (1994). Colonoscopy of acute colitis. A safe and reliable tool for assessment of severity. Dig Dis Sci. 39, 1550-1557.
18. Lémann M, Galian A, Rutgeerts P, Van H.R., Cortot A, Viteau J.M., et al. (1995). Comparison of budesonide and 5-aminosalicylic acid enemas in active distal ulcerative colitis. Aliment Pharmacol Ther. 9, 557–562.
19. Beattie, R. M., Nicholls S. W, Domizio P, Williams C. B., Walker-Smith J. A. (1996). Endoscopic Assessment of the Colonic Response to Corticosteroids in Children with Ulcerative Colitis. J Pediatr Gastroenterol Nutr. 22, 373-379.
20. Levine D.S., Riff D.S., Pruitt R, Wruble L, Koval G, Sales D, et al. (2002). A randomized, double blind, dose-response comparison of balsalazide (6.75 g), balsalazide (2.25 g), and mesalamine (2.4 g) in the treatment of active, mild-to-moderate ulcerative colitis. Am J Gastroenterol. 97, 1398–1407.
21. van Bergeijk J.D., Wilson J.H., Nielsen O.H., von Tirpitz C, Karvonen A.L., Lygren I, et al. (2002). OPUS 1 study group. Octreotide in patients with active ulcerative colitis treated with high dose corticosteroids (OPUS 1). Eur J Gastroenterol Hepatol. 14, 243-248.
22. Lindgren S, Löfberg R, Bergholm L, Hellblom M, Carling L, Ung KA, et al. (2002). Effect of budesonide enema on remission and relapse rate in distal ulcerative colitis and proctitis. Scand J Gastroenterol. 37, 705-710.
23. Rutter M, Saunders B, Wilkinson K, Rumbles S, Schofield G, Kamm M, et al. (2004). Severity of inflammation is a risk factor for colorectal neoplasia in ulcerative colitis. Gastroenterology. 126, 451-459.
24. Feagan B.G., Greenberg G.R., Wild G, Fedorak R.N., Paré P, McDonald J.W., et al. (2005). Treatment of Ulcerative Colitis with a Humanized Antibody to the α 4 β 7 Integrin. N Engl J Med. 352, 2499–2507.
25. Azzolini F, Pagnini C, Camellini L, Scarcelli A, Merighi A, Primerano AM, et al. (2005). Proposal of a new clinical index predictive of endoscopic severity in ulcerative colitis. Digestive Disease and Sciences. 50, 246‐251.
26. Nishio Y, Ando T, Maeda O, Ishiguro K, Watanabe O, Ohmiya N, et al. (2006). Pit patterns in rectal mucosa assessed by magnifying colonoscope are predictive of relapse in patients with quiescent ulcerative colitis. Gut. 55, 1768-1773.
27. Frøslie KF, Jahnsen J, Moum BA, Vatn MH; IBSEN Group. (2007). Mucosal healing in inflammatory bowel disease: results from a Norwegian population-based cohort. Gastroenterology. 133, 412-422.
28. Osada T, Ohkusa T, Okayasu I, Yoshida T, Hirai S, Beppu K, et al. (2008). Correlations among total colonoscopic findings, clinical symptoms, and laboratory markers in ulcerative colitis. Journal of Gastroenterology and Hepatology. 23. S262-S267.
29. Naganuma M, Ichikawa H, Inoue N, Kobayashi T, Okamoto S, Hisamatsu T, et al. (2010). Novel endoscopic activity index is useful for choosing treatment in severe active ulcerative colitis patients. J Gastroenterol. 45, 936-943.
30. Taro O, Toshifumi O, Tetsuji Y, Tomoyoshi S, Naoto S, Kazuko B, et al. (2010). Comparison of several activity indices for the evaluation of endoscopic activity in UC: Inter- and intraobserver consistency.Inflamm Bowel Dis. 16, 192-197.
31. Thia K.T., Loftus E.V. Jr, Pardi D.S., Kane S.V., Faubion W.A., Tremaine WJ, et al. (2011). Measurement of disease activity in ulcerative colitis: interobserver agreement and predictors of severity. Inflamm Bowel Dis. 17, 1257-1264.
32. Bessho R, Kanai T, Hosoe N, Kobayashi T, Takayama T, Inoue N, et al. (2011). Correlation between endocytoscopy and conventional histopathology in microstructural features of ulcerative colitis. J Gastroenterol. 46, 1197-1202.
33. Travis S.P., Schnell D, Krzeski P, Abreu M.T., Altman D.G., Colombel J.F., et al. (2012). Developing an instrument to assess the endoscopic severity of ulcerative colitis: the Ulcerative Colitis Endoscopic Index of Severity (UCEIS) Gut. 61, 535–542.
34. Kiesslich R, Duckworth C.A., Moussata D, Gloeckner A, Lim L.G., Goetz M, et al. (2012). Local barrier dysfunction identified by confocal laser endomicroscopy predicts relapse in inflammatory bowel disease. Gut. 61, 1146-1153.
35. Samuel S, Bruining DH, Loftus EV Jr, Thia KT, Schroeder KW, Tremaine WJ, et al. (2013). Validation of the ulcerative colitis colonoscopic index of severity and its correlation with disease activity measures. Clin Gastroenterol Hepatol. 11, 49–54.
36. Suzuki Y, Uchiyama K, Kato M, Matsuo K, Nakagawa T, Kishikawa H, et al. (2015). Potential utility of a new ulcerative colitis segmental endoscopic index combining disease severity and the extent of inflammation. J Clin Gastroenterol. 49, 401-406.
37. Lobaton T, Bessissow T, De H.G., Lemmens B, Maedler C, Van As.G, et al. (2015). The Modified Mayo Endoscopic Score (MMES): A New Index for the Assessment of Extension and Severity of Endoscopic Activity in Ulcerative Colitis Patients. J Crohns Colitis. 9, 846-852.
38. Kazuhiko U., Tomohisa T., Saori K., Yuki T., Makoto T., Yuma H., et al. (2017). Assessment of Endoscopic Mucosal Healing of Ulcerative Colitis Using Linked Colour Imaging, a Novel Endoscopic Enhancement System, Journal of Crohn's and Colitis. 11, 963-969.
39. Naganuma M, Yahagi N, Bessho R, Ohno K, Arai M, Mutaguchi M, et al. (2017). Evaluation of the severity of ulcerative colitis using endoscopic dual red imaging targeting deep vessels. Endosc Int Open. 5, E76-E82.
40. Iacucci M., Kiesslich R., Gui X.Y., Panaccione R, Heatherington J., Akinola O.,et al. (2017). Beyond white light: optical enhancement in conjunction with magnification colonoscopy for the assessment of mucosal healing in ulcerative colitis. Endoscopy. 49, 553-559.
41. Iacucci M, Daperno M, Lazarev M, Arsenascu R, Tontini GE, Akinola O, et al. (2017). Development and reliability of the new endoscopic virtual chromoendoscopy score: the PICaSSO (Paddington International Virtual ChromoendoScopy ScOre) in ulcerative colitis. Gastrointest Endosc. 86, 1118-1127.e5.
42. Chang J, Leong R.W., Wasinger V.C., Ip M, Yang M, Phan TG. (2017). Impaired Intestinal Permeability Contributes to Ongoing Bowel Symptoms in Patients With Inflammatory Bowel Disease and Mucosal Healing. Gastroenterology. 153, 723-731.e1.
43. Bálint A, Farkas K, Szepes Z, Nagy F, Szűcs M, Tiszlavicz L, et al. (2018). How disease extent can be included in the endoscopic activity index of ulcerative colitis: the panMayo score, a promising scoring system. BMC Gastroenterol. 18:7. doi: 10.1186/s12876-017-0725-3.
44. Hundorfean G, Chiriac M.T., Mihai S, Hartmann A, Mudter J, Neurath MF. (2017). Development and Validation of a Confocal Laser Endomicroscopy-Based Score for In Vivo Assessment of Mucosal Healing in Ulcerative Colitis Patients. Inflamm Bowel Dis. 24, 35-44.
45. Mine S, Takeshima F, Akazawa Y, Matsushima K, Minami H, Yamaguchi N, et al. (2018). Correlation of Fecal Markers with Magnifying Endoscopic Stratification in Patients with Ulcerative Colitis Who Are in Clinical Remission. Digestion. 97, 82-89.
46. Hosoe N, Nakano M, Takeuchi K, Endo Y, Matsuoka K, Abe T, et al. (2018). Establishment of a Novel Scoring System for Colon Capsule Endoscopy to Assess the Severity of Ulcerative Colitis-Capsule Scoring of Ulcerative Colitis. Inflamm Bowel Dis. 24, 2641-2647.
47. Rowan C.R., Cullen G, Mulcahy H.E., Sheridan J, Moss A.C., Ryan E.J., et al. (2019). DUBLIN (Degree of Ulcerative colitis Burden of Luminal Inflammation) score, a simple method to quantify inflammatory burden in ulcerative colitis. J Crohns Colitis. 13, 1365-1371.
48. Principi M, Contaldo A, Bianchi F.P., Losurdo G, Iannone A, Ierardi E, et al. (2020). Inter-Observer Agreement of a New Endoscopic Score for Ulcerative Colitis Activity: Preliminary Experience. Diagnostics (Basel). 10:213. doi: 10.3390/diagnostics10040213.
49. Bossuyt P, Nakase H, Vermeire S, de Hertogh G, Eelbode T, Ferrante M, et al. (2020). Automatic, computer-aided determination of endoscopic and histological inflammation in patients with mild to moderate ulcerative colitis based on red density. Gut. 69, 1778-1786.
50. Honzawa Y, Matsuura M, Higuchi H, Sakurai T, Seno H, Nakase H. (2020). A Novel endoscopic imaging system for quantitative evaluation of colonic mucosal inflammation in patients with quiescent ulcerative colitis. Endosc Int Open. 8, E41–E49.
51. Rahmi G, Coron E, Perrod G, Levy M, Moreau J, Moussata D, et al. (2021). Probe-based Confocal Laser Endomicroscopy for In Vivo Assessment of Histological Healing in Ulcerative Colitis: Development and Validation of the ENHANCE Index. J Crohns Colitis. 15, 994-999.
52. Liu L, Ouyang H, Su J, Lin Y, Hu Y, Shi H, et al. (2022). Increased modified DUBLIN scores are associated with serious ulcerative colitis and treatment failure. Therap Adv Gastroenterol. 15:17562848221142671. doi: 10.1177/17562848221142671.
53. Song Z, Zhang M, Ren Y, Iang B. (2022). [Improved Mayo Endoscopic Score has a higher value for evaluating clinical severity of ulcerative colitis]. Nan Fang Yi Ke Da Xue Xue Bao. 42, 997-1005.
54. Zittan E, Steinhart A.H., Aran H, Milgrom R, Gralnek I.M., Zelber-Sagi S, et al. (2022). The Toronto IBD Global Endoscopic Reporting [TIGER] Score: A Single, Easy to Use Endoscopic Score for Both Crohn's Disease and Ulcerative Colitis Patients. J Crohns Colitis. 16, 544-553.
55. Wan J, Wang X, Zhang Y, Xue X, Jia H, Wang M, et al. (2022). Time-adjusted average Mayo endoscopic score predicts the risk of disease extent progression in distal ulcerative colitis patients. Gastroenterol Rep (Oxf). 2022;10:goac019. doi: 10.1093/gastro/goac019.
56. Hayashi Y, Takabayashi K, Kato M, Tojo A, Aoki Y, Hagihara Y, et al. (2023). Usefulness of texture and color enhancement imaging in assessing mucosal healing in patients with ulcerative colitis. Gastrointest Endosc. 97, 759-766.e1.
57. Saito D, Hirai F, Uchiyama K, Takagi T, Naito Y, Takatsu N, et al. (2023). A new endoscopic scoring system corresponding to histological healing using linked color imaging in ulcerative colitis: the SOUL study. Endosc Int Open. 11, E504-E512.
58. Stidham R.W., Cai L, Cheng S, Rajaei F, Hiatt T, Wittrup E, et al. (2024). Using Computer Vision to Improve Endoscopic Disease Quantification in Therapeutic Clinical Trials of Ulcerative Colitis. Gastroenterology. 166, 155-167.e2.
59. Takabayashi K, Kobayashi T, Matsuoka K, Levesque BG, Kawamura T, Tanaka K, et al. (2023). Artificial intelligence quantifying endoscopic severity of ulcerative colitis in gradation scale. Dig Endosc. 36, 582-590.
60. Vitali F, Morgenstern N, Eckstein M, Atreya R, Waldner M, Hartmann A, et al. (2023). Endocytoscopy for assessing histologic inflammation in ulcerative colitis: development and prospective validation of the ELECT (ErLangen Endocytoscopy in ColiTis) score (with videos). Gastrointest Endosc. 97, 100-111.e1.
61. Truelove S.C., Richards W.C. (1956), Biopsy studies in ulcerative colitis. Br Med J. 1, 1315–1318.
62. Matts S.G. (1961). The value of rectal biopsy in the diagnosis of ulcerative colitis. Quarterly Journal of Medicine. 30, 393‐407.
63. Watts J.M., Thompson H, Goligher J/C. (1966). Sigmoidoscopy and cytology inthe detection of microscopic disease of the rectal mucosa in ulcerative colitis. Gut. 7, 288–294.
64. Keren D.F., Appelman H.D., Dobbins W.O., Wells J.J., Whisenant B, Foley J, et al. (1984). Correlation of histopathologic evidence of disease activity with the presence of immunoglobulin containing cells in the colons of patients with inflammatory bowel disease. Human Pathology. 15, 757–763.
65. Gomes P, du Boulay C, Smith CL, Holdstock G. (1986). Relationship between disease activity indices and colonoscopic findings in patients with colonic inflammatory bowel disease. Gut. 27, 92–95 .
66. Saverymuttu S.H., Camilleri M, Rees H, Lavender J.P., Hodgson H.J., Chadwick V.S. (1986). Indium 111‐granulocyte scanning in the assessment of disease extent and disease activity in inflammatory bowel disease. A comparison with colonoscopy, histology, and fecal indium 111‐granulocyte excretion. Gastroenterology. 90, 1121‐1128.
67. Floren C.H., Benoni C, Willen R. (1987). Histologic and colonoscopic assessment of disease extension in ulcerative colitis. Scandinavian Journal of Gastroenterology. 22, 459‐462.
68. Riley S.A., Mani V, Goodman M.J., Herd M.E., Dutt S, Turnberg L.A. (1988). Comparison of delayed‐release 5‐aminosalicylic acid (mesalazine) and sulfasalazine as maintenancetreatment for patients with ulcerative colitis. Gastroenterology. 94, 1383‐1389.
69. Riley SA, Mani V, Goodman MJ, Dutt S, Herd ME. (1991). Microscopic activity in ulcerative colitis: what does it mean? Gut. 32, 174–178.
70. Scheppach W, Sommer H, Kirchner T, Paganelli G.M., Bartram P, Christl S, et al. (1992). Effect of butyrate enemas on the colonic mucosa in distal ulcerative colitis. Gastroenterology. 103, 51-56.
71. Hanauer S, Schwartz J, Robinson M, Roufail W, Arora S, Cello J, et al. (1993). Mesalamine capsules for treatment of active ulcerative colitis: results of a controlled trial. Pentasa Study Group. American Journal of Gastroenterology. 88, 1188‐1197.
72. Odze R, Antonioli D, Peppercorn M, Goldman H. (1993). Effect of topical 5‐aminosalicylic acid (5‐ASA) therapy on rectal mucosal biopsy morphology in chronic ulcerative colitis. American Journal of Surgical Pathology. 17, 869‐875.
73. Geboes K., Riddell R., Ost A., et al. A reproducible grading scale for histological assessment of inflammation in ulcerative colitis. Gut. 2000;47(3):404–9.
74. Rutter M, Saunders B, Wilkinson K, Rumbles S, Schofield G, Kamm M, et al. (2004). Severity of inflammation is a risk factor for colorectal neoplasia in ulcerative colitis. Gastroenterology. 126, 451‐459.
75. Feagan B.G., Greenberg G.R., Wild G, Fedorak R.N., Paré P, McDonald J.W., et al. (2005). Treatment of Ulcerative Colitis with a Humanized Antibody to the α 4 β 7 Integrin. N Engl J Med. 352, 2499–2507.
76. Gupta RB, Harpaz N, Itzkowitz S, Hossain S, Matula S, Kornbluth A, et al. (2007). Histologic inflammation is a risk factor for progression to colorectal neoplasia in ulcerative colitis: a cohort study. Gastroenterology. 133, 1099–1105. 3
77. Gramlich T, Petras R.E. (2007). Pathology of inflammatory bowel disease. Seminars in Pediatric Surgery. 16, 154‐163.
78. Baars J.E., Nuij J.A.A., Oldenburg B, Kuipers E.J., van der Woude C.J. (2012). Majority of patients with inflammatory bowel disease in clinical remission have mucosal inflammation. Inflammatory Bowel Diseases. 18, 1634‐1640.
79. Rubin D.T., Huo D, Kinnucan J.A., Sedrak M.S., McCullom N.E., Bunnag A.P., et al. (2013). Inflammation is an independent risk factor for colonic neoplasia in patients with ulcerative colitis: a case-control study. Clin Gastroenterol Hepatol. 11, 1601-1608.e1-4.
80. Iacucci M, Fort G.M., Hassan C, Panaccione R, Kaplan G.G., Ghosh S, et al. (2015). Complete mucosal healing defined by endoscopic Mayo subscore still demonstrates abnormalities by novel high definition colonoscopy and refined histological gradings. Endoscopy. 47, 726-734.
81. Theede K, Holck S, Ibsen P, Ladelund S, Nordgaard-Lassen I, Nielsen A.M. (2015). Level of Fecal Calprotectin Correlates With Endoscopic and Histologic Inflammation and Identifies Patients With Mucosal Healing in Ulcerative Colitis. Clin Gastroenterol Hepatol. 13, 1929–1936.e1.
82. Zenlea T, Yee E.U., Rosenberg L, Boyle M, Nanda K.S., Wolf J.L., et al. (2016). Histology Grade Is Independently Associated With Relapse Risk in Patients With Ulcerative Colitis in Clinical Remission: A Prospective Study. Am J Gastroenterol. 111, 685-690.
83. Aranzazu J.A, Auke G, Yannick D, Bart L, Xavier S, Talat B, Tet al. (2017). A Simplified Geboes Score for Ulcerative Colitis, Journal of Crohn's and Colitis. 11, 305-313.
84. Marchal-Bressenot A, Salleron J, Boulagnon-Rombi C, Bastien C, Cahn V, Cadiot G, et al. (2017). Development and validation of the Nancy histological index for UC. Gut. 66, 43–49.
85. Mosli M.H., Feagan B.G., Zou G, Sandborn W.J., D'Haens G, Khanna R, et al. (2017). Development and validation of a histological index for UC. Gut. 66, 50-58.
86. Christensen B, Hanauer S.B., Erlich J, Kassim O, Gibson P.R., Turner J.R., et al. (2017). Histologic Normalization Occurs in Ulcerative Colitis and Is Associated With Improved Clinical Outcomes. Clin Gastroenterol Hepatol. 15, 1557-1564.e1.
87. Caputo A, Parente P, Cadei M, Fassan M, Rispo A, Leoncini G, et al. (2022). Simplified Histologic Mucosal Healing Scheme (SHMHS) for inflammatory bowel disease: a nationwide multicenter study of performance and applicability. Tech Coloproctol. 26, 713-723.
88. Villanacci V, Antonelli E, Lanzarotto F, Bozzola A, Cadei M, Bassotti G. (2017). Usefulness of Different Pathological Scores to Assess Healing of the Mucosa in Inflammatory Bowel Diseases: A Real Life Study. Sci Rep. 7:6839. doi: 10.1038/s41598-017-07338-x.
89. Jangi S, Yoon H, Dulai P.S., Valasek M, Boland B.S., Jairath V, et al. (2020). Predictors and outcomes of histological remission in ulcerative colitis treated to endoscopic healing. Aliment Pharmacol Ther. 52, 1008-1016.
90. Lang-Schwarz C, Angeloni M, Agaimy A, Atreya R, Becker C, Dregelies T, et al. (2021). Validation of the 'Inflammatory Bowel Disease-Distribution, Chronicity, Activity [IBD-DCA] Score' for Ulcerative Colitis and Crohn´s Disease. J Crohns Colitis. 15, 1621-1630.
91. Gui X, Bazarova A, Del Amor R, Vieth M, de Hertogh G, Villanacci V, et al. (2022). PICaSSO Histologic Remission Index (PHRI) in ulcerative colitis: development of a novel simplified histological score for monitoring mucosal healing and predicting clinical outcomes and its applicability in an artificial intelligence system. Gut. 71, 889-898.
